# Silicon Spike: An Arduino-based low-cost and open-access triggerbox to precisely control TMS devices

**DOI:** 10.3758/s13428-025-02653-y

**Published:** 2025-04-15

**Authors:** Giuseppe Ippolito, Thomas Quettier, Sara Borgomaneri, Vincenzo Romei

**Affiliations:** 1 Center for Studies and Research in Cognitive Neuroscience, Department of Psychology “Renzo Canestrari”, Cesena Campus, Alma Mater Studiorum, Università Di Bologna, 47521 Cesena, Italy; 2 Laboratory of Cognitive Neuroscience, Department of Languages and Literatures, Communication, Education and Society, University of Udine, Udine, Italy; 3 Universidad Antonio de Nebrija, Madrid, Spain

**Keywords:** TMS, rTMS, ccPAS, Triggerbox, Arduino, Open source

## Abstract

Transcranial magnetic stimulation (TMS) is a widely used tool in the field of clinical and cognitive neuroscience. To exploit its excellent temporal properties, TMS usually relies on triggerbox devices, which temporize the delivery of magnetic pulses according to the paradigm requirements. However, a main limitation of most of the widely used triggerbox devices is that they rely solely on the experimental computer processor, which might add temporal uncertainty in delivering the TMS pulse when the computer’s resources are drained by other experimental devices or by task execution itself, especially during repetitive TMS or dual-coil protocols. We aimed at developing a low-cost and easily reproducible triggerbox device which could overcome these limitations by relying on an external processor to handle the timing precision. We used an Arduino Uno R4 Minima to build Silicon Spike, a low-cost ($60) triggerbox device. We tested the device’s precision in delivering the TMS pulses under different working load conditions, and the impact over time. All of the tests were ecological, delivering real TMS pulses during dual-coil, repetitive, and patterned TMS protocols. We obtained extremely high precision (< 0.022 ms) in all of the tests. This means that, for smaller or longer latencies, the error remains negligible for TMS studies. Thus, the Silicon Spike device demonstrated microsecond precision in handling the TMS pulse delivery, establishing itself as a simple and yet precise device. We freely provide the source code and the hardware schematics, allowing anyone to reproduce our work.

**Supplementary Information:**

The online version contains supplementary material available at 10.3758/s13428-025-02653-y.

## Introduction

Studying the “when” of neurophysiological processes implies a fine-grained time resolution of the investigation tools employed. Indeed, most cognitive events take place in a matter of milliseconds; thus, a high level of precision is necessary when presenting stimuli or harnessing experimental manipulations. In order to accomplish this, a vast range of software has been developed (e.g., MATLAB Psychtoolbox, E-Prime, PsychoPy, OpenSesame). Most of these programs allow one to create and present the task stimuli according to the experimental hypothesis with high timing precision while recording the participant’s response. However, as usually happens in neurophysiological research, several other devices are typically involved for recording or manipulating the participant’s behavior or other physiological variables. Consequently, their proper, coordinated handling is crucial when building a new task, in order to use them without harming the task precision. This is the case with transcranial magnetic stimulation (TMS), in which a brief magnetic pulse is delivered to the participant’s scalp to noninvasively modulate their brain functioning (Hallett, 2007). This approach requires close attention to the code structure in order to trigger the device without causing any delay in the experimental task. This is particularly relevant in paired-pulse or dual-coil protocols (e.g., cortico–cortical paired associative stimulation [ccPAS]), or rhythmic and repetitive TMS (rTMS) protocols, in which multiple TMS pulses are delivered to modulate the brain’s activity, usually with extremely short intervals—even under 10 ms (Hernandez-Pavon et al., 2023; Di Luzio et al., 2024; Tarasi et al., 2024; Ippolito et al., 2022; Klomjai et al., 2015; Trajkovic et al., 2022; 2024a, b)—or concomitantly with the stimulus onset (Bertaccini et al., 2023).

In most cases, the communication between TMS devices and the experimental computer is handled through a triggerbox device, an electronic apparatus that allows the TMS to be triggered when a specific prompt is run. However, triggering TMS pulses using the same computer engaged in the task execution—which might involve the presentation of auditory, somatosensory, and/or visual stimuli, response collection, or even communication between several other experimental devices (Borgomaneri et al., 2023; Fotia et al., 2021; Trajkovic et al., 2022)—might result in a delay in the pulse emission. This is particularly relevant for dual-coil TMS and rTMS protocols, in which the sum of small latencies might exceed the computer’s resources, leading to an imprecise calculation of distances between the TMS pulses and thus impacting the experimental results. This risk can be avoided by allocating this kind of computation to an external processor, such as that of the triggerbox, allowing for finer timing implementation. Still, most triggerbox devices rely solely on the computer resources. Moreover, the principle behind the triggerbox devices is quite simple, from both a hardware and software point of view. Consequently, it is common to come across handmade reproductions using simple components. Specifically within the electronic and programming community, Arduino products (see: https://www.arduino.cc/) have been distinguished for their accessible price, ease of use, and completely open-access availability (Ismailov & Jo, 2022; Kondaveeti et al., 2021). This has led to a flourishing market and numerous projects shared by enthusiasts and professionals, facilitating their replication across different labs (White et al., 2019). Nonetheless, tests conducted on Arduino’s latencies during different tasks with variable code structure and workload (D’Ausilio, 2012) have demonstrated how these cheap machines can provide reliable performance and extremely accurate timing, making them suitable for the lab setting. For this reason, they have been implemented in a multitude of experiments including real-time wireless electrocardiogram (ECG) (Güvenç, 2020), light-emitting diode (LED) stimulators for visual research (Teikari et al., 2012), or even drowsiness sensors based on EEG signals (Mindoro, 2020).

**Fig. 1:**
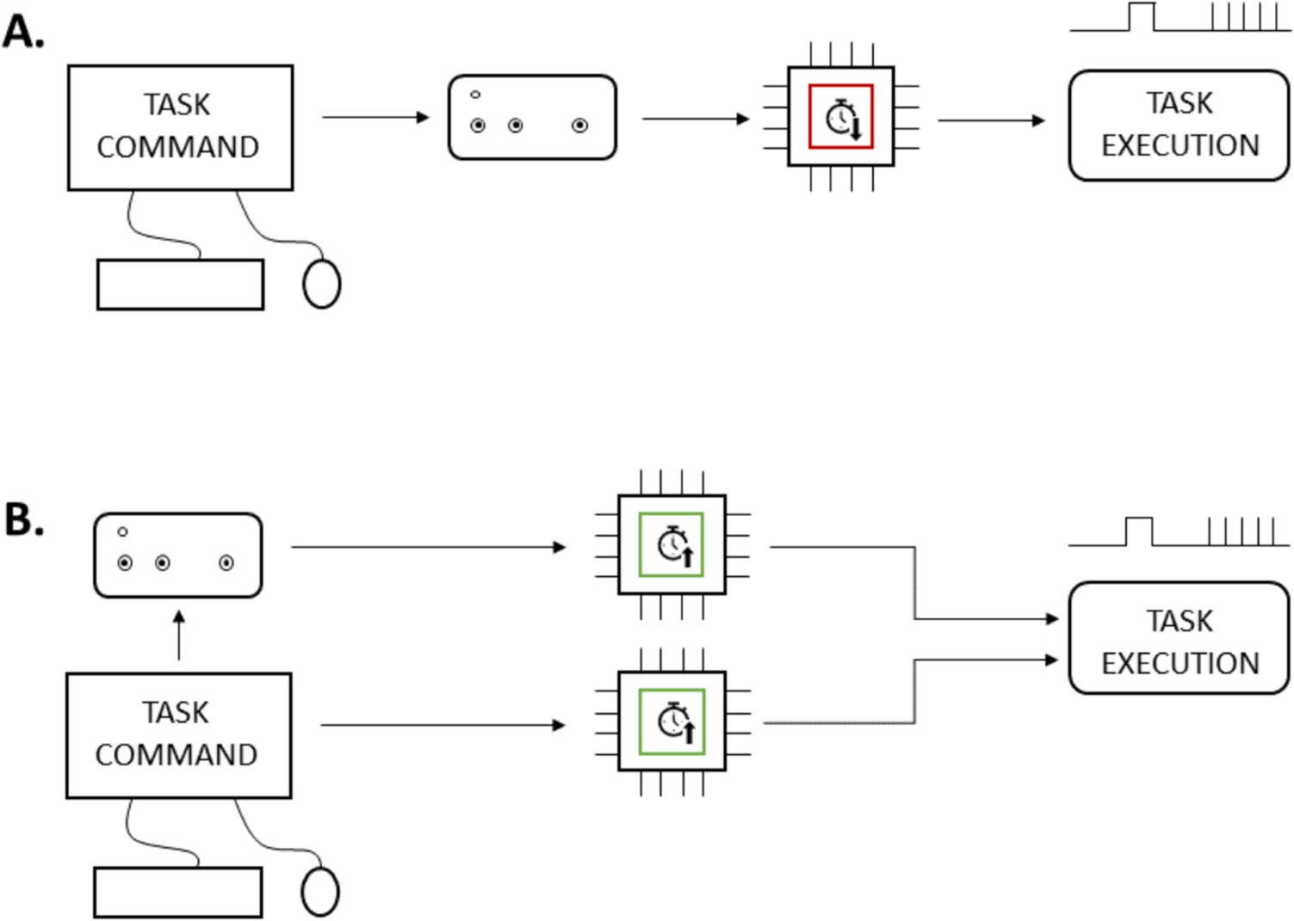
Visual representation of the difference between a single- (**A**) and multi-processor (**B**) approach. Using the former, both the task and TMS timings rely on the computer’s processor, potentially adding latencies. The latter protocol instead leaves the TMS timings to the triggerbox, allowing the computer to allocate its resources solely to the experimental task

Given this versatility and reliability, it is common to find an Arduino-based triggerbox device in several labs employing TMS. This allows them to run several experiments simultaneously without the need to buy commercially available triggerbox devices, whose cost is estimated to be on average around **€**1000 each. Nevertheless, custom-made triggerbox devices generally share the same limitations as commercially available systems: they rely on the computer’s processor to handle timings (Fig. 1). In other words, they are used merely as a passive transfer to transform a serial input from the experimental computer into a square wave (i.e., transistor–transistor logic [TTL]) to trigger the TMS pulses. As stated earlier, such an approach only allows for establishing communication between the experimental computer and the TMS, thus allocating the resources for handling the TMS trigger to the computer itself rather than to the triggerbox. In other words, the triggering precision might be reduced during high-demand protocols (Joao et al., 2012), such as rTMS/dual-coil or even continuous (cTBS) or intermittent (iTBS) theta-burst protocols. Additionally, a few software programs, such as PsychoPy (Peirce et al., 2019), use the screen refresh rate as a standard unit to handle timings. This means that the interval between two actions must necessarily be a multiple of the refresh rate. Considering that most lab computers run at a frequency of 100 Hz—or, occasionally, at 60 Hz—only one action can be performed every 10 ms—or 16.67 ms—making it impossible to stimulate during ccPAS/rTMS protocols with an inter-pulse interval different from the refresh rate multiples.

An alternative approach, as we propose here, involves using the Arduino processor to handle two pulses and the distance between them, given a simple computer prompt. This enables totally independent control of TMS timing, lightening the computer resources. This should ease the task execution and the triggering rate precision. When possible, this approach is preferable (Krauss, 2020).

However, this approach usually requires programming of the Arduino’s motherboard for each of the stimulation parameters according to the task demands. This implies that programming skills are required, and that the continuous updating of the code might be more prone to error. This is particularly worrisome when several users harness the lab settings or when numerous experimental setups are needed in the lab, meaning that the device needs on-demand coding updates, even several times a day. Alternatively, it is possible to write more complex and flexible codes which reduce the number of times the script needs to be modified. But this also implies that more exhaustive tests on its precision are necessary. To the best of our knowledge, there has been no publication to date in which a TMS triggerbox was built using this specific approach.

**Fig. 2:**
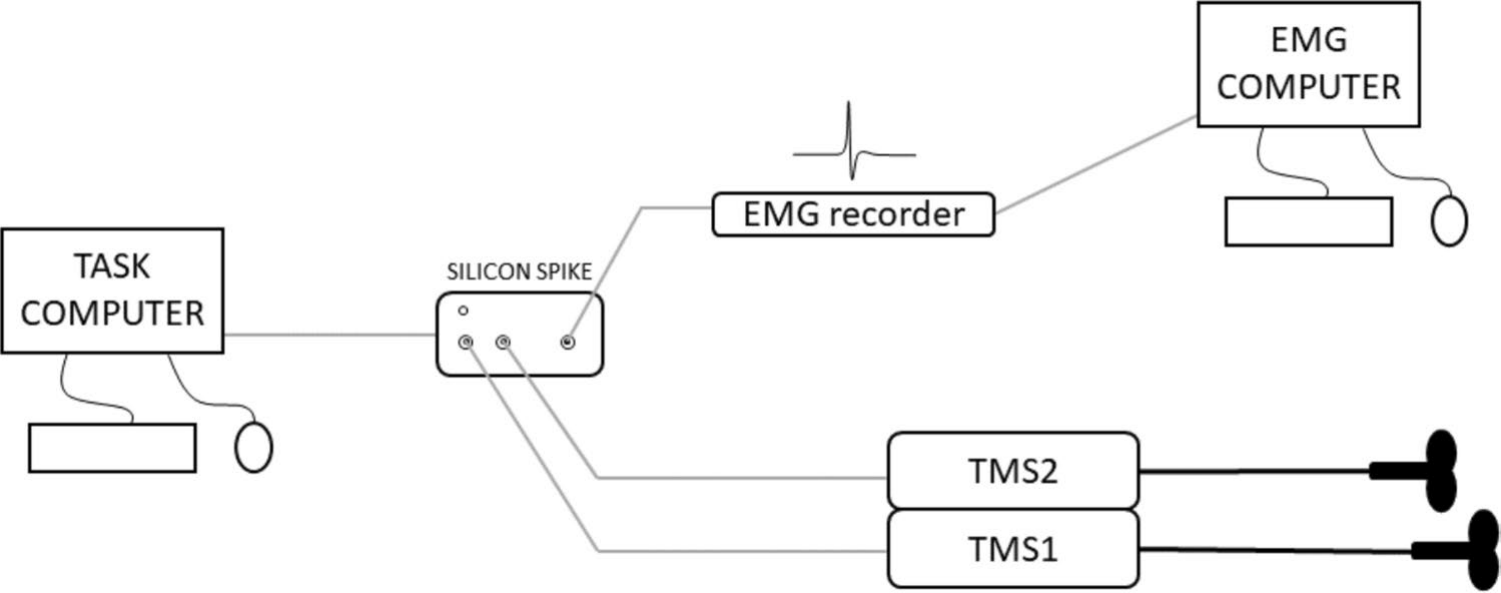
Schematization of a classical lab configuration. In this case the task computer gives an input to the Silicon Spike triggerbox, which autonomously coordinates TMS and EMG timings and markers, thus not relying on the computer processor. This resource allocation allows a smoother execution of the experimental task

In order to overcome these limitations, given the higher precision of the multiprocessor versus single-processor approach, we developed a triggerbox device which relies on an additional processor to easily and reliably handle TMS pulse delivery (Fig. 2). Here we test the device’s precision in delivering TMS pulses during single-pulse (spTMS), dual-coil, and rTMS protocols, each under different load conditions. The final product allows the user to implement our solution even with little or no programming knowledge. Thus, the aim of the present study is to introduce the Silicon Spike device and working principles to the community of TMS users, to enable the reproduction of the circuit and free use of the device.

## Silicon Spike device

**Fig. 3:**
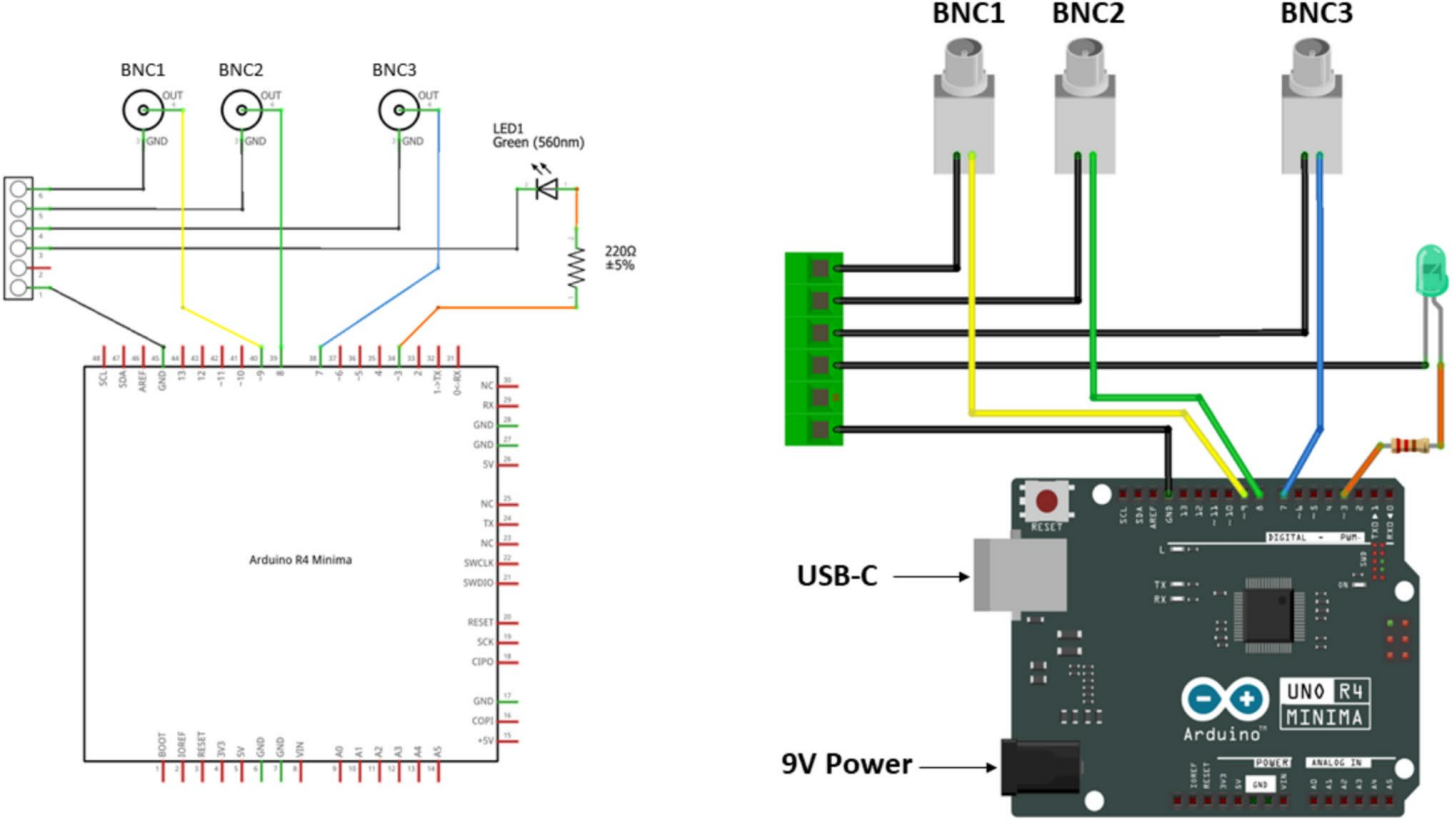
Silicon Spike hardware in **a** its schematic circuit and **b** graphical representation. Full details in Supplementary Materials or here: https://ippoz.gitbook.io/siliconspiketriggerbox/

Here we present an Arduino-based custom-made device whose production cost is currently around **€**60. This device consists of a hardware part (Fig. 3) and its proper code (available here: https://github.com/Ippolz/SiliconSpike), which needs to be uploaded once on the device motherboard. All of the necessary stimulation parameters (e.g., number of pulses and the distance between them) used for spTMS, rTMS, ccPAS, cTBS, or iTBS can consequently be established through common software used in the research field, such as MATLAB or Python among others. Due to their length and technical specificity, assembly instructions are covered in its user manual (available here: https://ippoz.gitbook.io/siliconspiketriggerbox/; also see Supplementary Materials). The Silicon Spike hardware consists of the following:

- Arduino Uno R4 Minima (https://store.arduino.cc/products/uno-r4-minima)
- Three BNC pins (TTL outputs)
- One LED and its proper resistor
- One USB type-C (serial communication)
- One 9 V 2.1 mm power jack

The code structure allows for the use of the same software (e.g., MATLAB) running the task to control the stimulation parameters. Once the serial communication is established, the experimenter can define the following:

- The inter-pulse interval (IPI), consisting of the distance between each pulse (only for rTMS and dual-coil protocols)
- The number of pulses within each train (only for rTMS protocols)
- The length of a square wave (5 V), to use it as a marker in the absence of any TMS pulse (e.g., stimulus onset)

All these parameters can be independently declared for nine presets within the same stimulation protocol (e.g., a dual-coil TMS with nine different distances between pulses to call separately in the same task). After that, you need to specify which protocol to use (spTMS, rTMS, dcTMS). Note that iTBS and cTBS protocols are extensions of the rTMS one, whereas the ccPAS is an extension of the dual-coil protocol.

## Materials and methods

**Fig. 4:**
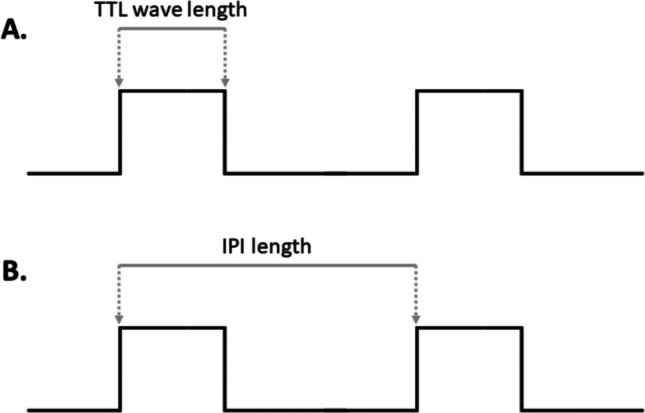
The two main measures we used are **A** the TTL wavelength, which is the duration of the digital square wave, and **B** the IPI length, which is the distance between the beginning of a digital wave and the consecutive one

In order to measure the precision of the Silicon Spike device timing, we used a Biopac MP-35 (BIOPAC Systems Inc., USA) system and BSL Analysis 4.1 software. Eight tests were conducted to measure the device performance under different load conditions for both rTMS and ccPAS (results also apply to spTMS, being simpler and derived from the previous ones), using a MATLAB (version R2022a) script (available here: https://github.com/Ippolz/SiliconSpike) consisting of a loop containing the specific protocol parameters and the firing commands. Each pulse produced a digital square wave of 5 V amplitude on the Biopac recording, running at a 20,000 Hz sampling rate. In order to mimic a realistic scenario, the Silicon Spike device was also connected to the TMS machine, which delivered TMS pulses at 50% of the maximum stimulator output intensity. For the dual-coil protocol, a Magstim® BiStim^2^ model was used, while for the rTMS and iTBS/cTBS protocols, a Magstim® Rapid^2^ was used. The marker (TTL) length (in tests 2, 4, 5) and the interval between each pulse (IPI, in tests 1, 2, 3, 4, 5, 6, 7) were measured (Fig. 4).

### Test 1: Low-load rTMS

The first test investigates the rTMS protocol in a low-load condition, delivering 100 trains of seven pulses each, with an IPI of 100 ms (10 Hz) and an interval of 1 s between each train start. Thus, we measured the device precision for each IPI (600 trials).

### Test 2: High-load rTMS

The second test repeats the previous one in a high-load condition, creating four different presets for the rTMS protocol. Thus, 100 trains of seven pulses each were delivered for each different IPI (70, 90, 120, 150 ms), randomly called with a distance of 2 s. Each of these is preceded by a random marker between the four declared in the setting phase (7, 9, 12, 15 ms) and a 1 s pause. The TTL length (100 × 4 = 400 trials) and the IPI distance (600 × 4 = 2400 trials) were considered.

### Test 3: Low-load ccPAS

This test is aimed at measuring the precision of the ccPAS protocol during a low-load condition. Thus, a series consisting of 100 pairs of pulses with a distance of 150 ms between them is presented with a 1 s interval. The TTL length (100 trials) was measured.

### Test 4: High-load ccPAS (long IPIs)

The fourth test repeats the previous one in a high-load condition, creating four different presets for the ccPAS protocol. In this case, long IPIs (150, 500, 700, 1000 ms) were used, with an interval of 4 s between each pair. Each of these pairs was presented randomly, and anticipated by a random marker of a chosen length (7, 10, 15, 20 ms). The TTL length (100 × 4 = 400 trials) and the IPI distance (100 × 4 = 400 trials) were measured.

### Test 5: High-load ccPAS (short IPIs)

The fifth test mimics the fourth one, presenting four pulse pairs preceded by a marker but using short IPIs (7, 10, 15, 20 ms) instead. The TTL length (100 × 4 = 400 trials) and the IPI distance (100 × 4 = 400 trials) were measured.

### Test 6: iTBS

The sixth test uses the rTMS parameters to reproduce an iTBS protocol, delivering brief bursts of three pulses each at 50 Hz repeated every 200 ms in short trains of 2 s each repeated every 10 s (20 trains, 600 pulses). The IPI distance (400 trials) was measured.

### Test 7: cTBS

The seventh test reproduces a cTBS protocol, delivering brief bursts of three pulses each at 50 Hz repeated every 200 ms for 20 s (300 pulses). The IPI distance (200 trials) was measured.

### Test 8: IPI length effect

The eighth and last test is aimed at assessing whether there is an accumulation of latencies at increasing times, by delivering ccPAS pulses whose IPIs increase linearly from short (10 ms) to long (800 ms) intervals. Each interval (10, 100, 200, 300, 400, 500, 600, 700, 800 ms) was repeated 100 times. The IPI distance (800 trials) was measured.

## Results

In seven separate tests, we measured the temporal precision for the interval between each pulse (IPI), while in tests 2, 4, and 5 we measured the temporal precision of the marker (TTL) length. For both indices we took into consideration the average variability in the wave duration (standard deviation) and the difference between the expected and the actual duration (delay). Overall, we observed very little difference between the expected and the effective durations (see Table 1). By considering the TTL wavelength, we registered an average delay of 0.008 ms (min = 0.005 ms, max = 0.011 ms). The oscillation around the expected duration was 0.018 ms (min = 0.016 ms, max = 0.021 ms). Results were similar when considering the IPI performance, with an average delay of 0.006 ms (min = –0.011 ms, max = 0.011 ms) between the expected and the effective interval, with a 0.018 ms (min = 0.009 ms, max = 0.021 ms) oscillation around the expected value.

**Table 1:** Average data resulting from the 28 simulations obtained from tests 1 to 7

	**Test 1—TTL length**				**Test 1—IPI length**			
**Expected duration (ms)**	–	–	–	–	100	–	–	–
**Avg (ms)**	–	–	–	–	100.010	–	–	–
**SD (ms)**	–	–	–	–	0.020	–	–	–
**Delay (ms)**	–	–	–	–	0.010	–	–	–
	**Test 2—TTL length**				**Test 2—IPI length**			
**Expected duration (ms)**	7	9	12	15	70	90	120	150
**Avg (ms)**	7.007	9.008	12.006	15.010	70.010	90.011	120.009	150.009
**SD (ms)**	0.017	0.019	0.016	0.020	0.020	0.020	0.019	0.019
**Delay (ms)**	0.007	0.008	0.005	0.010	0.010	0.011	0.009	0.009
	**Test 3—TTL length**				**Test 3—IPI length**			
**Expected duration (ms)**	–	–	–	–	150	–	–	–
**Avg (ms)**	–	–	–	–	150.005	–	–	–
**SD (ms)**	–	–	–	–	0.016	–	–	–
**Delay (ms)**	–	–	–	–	0.004	–	–	–
	**Test 4—TTL length**				**Test 4—IPI length**			
**Expected duration (ms)**	7	10	15	20	150	500	700	1000
**Avg (ms)**	7.006	10.008	15.007	20.008	150.006	500.000	699.999	999.989
**SD (ms)**	0.016	0.018	0.017	0.018	0.018	0.011	0.009	0.021
**Delay (ms)**	0.005	0.007	0.006	0.008	0.006	–0.001	–0.001	–0.011
	**Test 5—TTL length**				**Test 5—IPI length**			
**Expected duration (ms)**	7	10	15	20	7	10	15	20
**Avg (ms)**	7.009	10.006	15.011	20.007	7.007	10.008	15.008	20.011
**SD (ms)**	0.019	0.016	0.021	0.017	0.017	0.018	0.018	0.020
**Delay (ms)**	0.009	0.005	0.011	0.006	0.007	0.007	0.007	0.010
	**Test 6—TTL length**				**Test 6—IPI length**			
**Expected duration (ms)**	–	–	–	–	10	–	–	–
**Avg (ms)**	–	–	–	–	10.012	–	–	–
**SD (ms)**	–	–	–	–	0.021	–	–	–
**Delay (ms)**	–	–	–	–	0.012	–	–	–
	**Test 7—TTL length**				**Test 7—IPI length**			
**Expected duration (ms)**	–	–	–	–	10	–	–	–
**Avg (ms)**	–	–	–	–	10.012	–	–	–
**SD (ms)**	–	–	–	–	0.022	–	–	–
**Delay (ms)**	–	–	–	–	0.012	–	–	–

Values are indicative of the device precision in producing a marker of a fixed duration (TTL length) or a series of TTL waves with a fixed interval (IPI length). Here, for each condition, we can see the expected duration, which is the input value, the average (the average TTL wavelength), SD (standard deviation), and delay (the difference in time between the TTL wave duration and the input value).

**Fig. 5:**
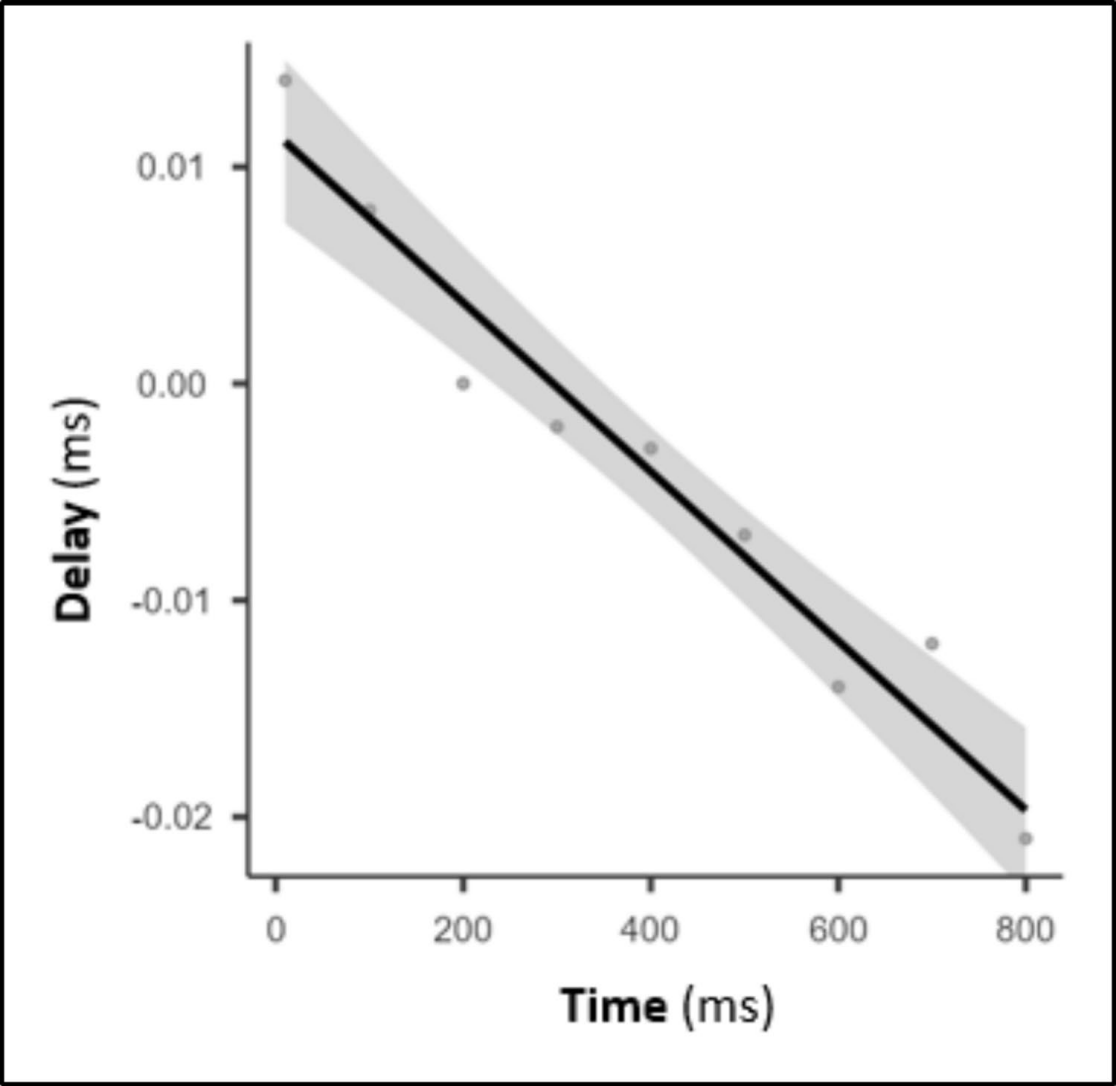
Linear regression analysis between time and delay. With increasing time, the delay is reduced

In the eighth test, we investigated whether using greater IPIs would cause longer delays. Thus, we used a ccPAS paradigm whose IPIs ranged evenly between 10 and 800 ms, measuring its delay. The linear regression analysis (Fig. 5) revealed a significant negative relation (*R* = 0.988, *p* < 0.001), suggesting that the delay is progressively reduced at increasing times, becoming negative over approximately 300 ms. However, despite being significant, the delay range (from –0.0195 to 0.0095 ms) is over 50 times smaller than a millisecond, suggesting that this latency does not negatively affect TMS results.

## Discussion

TMS is a widely used technique both in clinical settings and in the field of cognitive neuroscience. It makes it possible to selectively interact with one or more brain regions with a neural process by perturbing or even enhancing (Bertaccini et al., 2023; Borgomaneri et al., 2023; Di Gregorio et al., 2022; Trajkovic et al., 2022; 2024a, b) their function, for example, during an experimental task. Many TMS protocols can significantly manipulate the ongoing brain activity by employing time-sensitive interventions which require precise timing delivery for optimal synchronized stimulus presentation and effective communication between stimulation devices. In this respect, the communication between the experimental computer and the TMS device is usually handled through the use of commercially available triggerbox devices, which simply translate the inputs into triggering instructions without relying on a dedicated internal clock. Indeed, this kind of approach is notoriously prone to bottleneck overflow (Joao et al., 2012), which might end in unwanted stimulus presentation latencies, especially in complex tasks or multi-pulse TMS (such ccPAS, rTMS, or iTBS/cTBS) protocols, since most of them are not provided with built-in functions for delivering pulses in series. This risk can be avoided by relying on an external processor to handle timings, in order to keep the computations related to the TMS triggering completely independent from those performed by the experimental computer (i.e., electromyographic recordings, EEG recordings, skin conductance, eye tracker, heart rate monitoring, stimuli presentation, response collection). However, a similar approach requires more complex programming, since several aspects specific to each TMS protocol must be considered. This means that relying on a dedicated processor affords greater precision, but is also potentially more prone to error. Here, we addressed this point by building Silicon Spike, a triggerbox device specifically designed to precisely trigger TMS devices for most of its stimulation protocols (i.e., spTMS, dcTMS, ccPAS, rTMS, rhTMS, iTBS, cTBS). The hardware consists of an Arduino Uno R4 Minima and a few other small components, which makes it a highly precise yet low-cost device (around **€**60). We freely provide the hardware schematics and the source code (https://github.com/Ippolz/SiliconSpike), meaning that the device can be easily assembled, and it does not need to be programmed. Also, its instructions are covered in detail (see Supplementary Materials or https://ippoz.gitbook.io/siliconspiketriggerbox/). We are confident that this approach will increase its ease of use and will encourage colleagues to improve our work by adapting the Silicon Spike device according to the constantly changing needs of the research field.

In this paper we aimed at measuring the precision of the Silicon Spike under a series of realistic TMS protocols with various degrees of computational load. Our results show that the device is highly reliable, with an oscillation of just a few microseconds, across the different loading conditions. Further, we assessed whether these delays, however small, might accumulate at increasing times. Thus, we conducted an additional test in which we delivered pulse pairs at progressively longer distances, measuring their delay. Results indicate that, for small (10 ms) to long (800 ms) distances, the delay is a fraction dozens of times smaller than a millisecond. This means that the Silicon Spike device can be used by anyone for TMS research. It is important to stress the fact that, unlike many commercially available triggerbox devices, Silicon Spike is built on the concept that any temporal computation for stimulus delivery within and between devices relies on the external processor circuitry (the Silicon Spike in particular). This advantage allows the TMS triggering precision to be effectively unaffected by the execution of concurrent experimental task loads, or any other device timestamp requirement involved in the data collection. We believe that this aspect is crucial in the lab setting, where several devices are often involved in the data collection. Also, the Silicon Spike device allows one to set up to nine stimulation protocols at once (e.g., rhTMS at 10 or 11 Hz; or ccPAS at 50, 70, or 120 ms). This means that it is possible to shuffle them within a single experimental block, enabling a finer trial randomization.

It might be argued that we do not directly compare our approach, which consists in parallel processing of instructions, to the approach used by most commercially available triggerbox devices, in which inputs are sent in series. However, such a comparison would be beyond the scope of this work. Indeed, commercial devices can be heterogeneous for both the hardware and programming part, often being built for a few specific purposes. In this view, we thought a comparison between devices might not be necessary. What we do know is that the Silicon Spike device has precision far above the millisecond in delivering TMS pulses; thus, we can affirm that it can be safely used in cognitive neuroscience practice.

Embracing an open-science framework, we provide the scripts we used to collect the present data (https://github.com/Ippolz/SiliconSpike) so that it is possible to replicate our exact analysis or extend it to include new parameters. As the entire project is freely available online, we are confident that it can be modified and improved by our colleagues in the scientific community. When we built the Silicon Spike device, we aimed at making it as easy as possible to reproduce, so we opted for a compact and simple design. Still, we think that it is possible, through derivative designs, to build a triggerbox tuned for some specific stimulation paradigms which we do not currently cover (e.g., three-coil TMS), thus overcoming current limitations. Therefore, we highly encourage readers to improve upon our work. Colleagues are also allowed to take advantage of our scripts in order to reproduce our reliability tests on their alternative versions of the Silicon Spike device.

## Supplementary Information

Below is the link to the electronic supplementary material.

Supplementary file1 (DOCX 529 KB)

## Data Availability

Data are available at: https://github.com/Ippolz/SiliconSpike.

## Code availability

Code available at: https://github.com/Ippolz/SiliconSpike.

## Declarations

### Open Practices

Stimuli, validation data, and analysis code are available at: https://github.com/Ippolz/SiliconSpike.
