# Supplementary material for: Silicon Spike: An Arduino-based low-cost and open-access triggerbox to precisely control TMS devices
Source: Behav Res Methods. 2025 Apr 15;57(5):145. doi: 10.3758/s13428-025-02653-y (PMC12000144; doi:10.3758/s13428-025-02653-y)
Supplement: Supplementary file 1 — Supplementary file1 (DOCX 529 KB) [file 13428_2025_2653_MOESM1_ESM.pdf]

SILICON SPIKE

USER MANUAL

SILICON SPIKE

GIUSEPPE IPPOLITO
&
THOMAS QUETTIER

DOI: 10.3758/s13428-025-02653-y

Original work

Please, for any question about performance and timing reliability refer to the original publication:

Ippolito G., Quettier T., Borgomaneri S., Romei V. (2025). Silicon Spike: an Arduino-based low-cost and open-access triggerbox to precisely control TMS devices. *Behavior Research Methods*. https://doi.org/10.3758/s13428-025-02653-y

DOI: https://doi.org/10.3758/s13428-025-02653-y

Contacts: giuseppe.ippolito8@unibo.it

Introduction

Here we report a novel and reliable tool to trigger transcranial magnetic stimulation (TMS) devices with almost null latencies, easing the task execution during lab experiments. This goal has been achieved with exceptionally good results. Hence, we decided to make the Silicon Spike triggerbox a freely accessible device for anyone to reproduce, implement, and share. If you are using the Silicon Spike triggerbox in your experiment, please acknowledge our work (https://doi.org/10.3758/s13428-025-02653-y).

Relative to the most commonly available triggerbox devices, the advantage of Silicon Spike consists in leaving all of the computations necessary to trigger the TMS to its internal motherboard, without interfering with the computer executing the experimental task. It allows control of all the stimulation parameters for the single pulse (spTMS), repetitive/rhythmic (rTMS), and dual coil (dcTMS/ccPAS) protocols with few lines of code, also making it accessible for those without any programming knowledge. It is also possible to set the rTMS parameters to obtain a continuous (cTBS) or intermittent (iTBS) theta-burst stimulation. The stimulation parameters can be declared using any software allowing serial communication; here we will cover in detail this procedure using MATLAB and Python.

Hardware

The Silicon Spike device is composed of the following elements:

- Arduino Uno R4 Minima (product details here: https://store.arduino.cc/products/uno-r4-minima);
- Three BNC pins;
- One LED and its proper resistor;
- One USB type-C;
- One 9 Volt 2.1mm power jack

These components need to be assembled following this scheme:

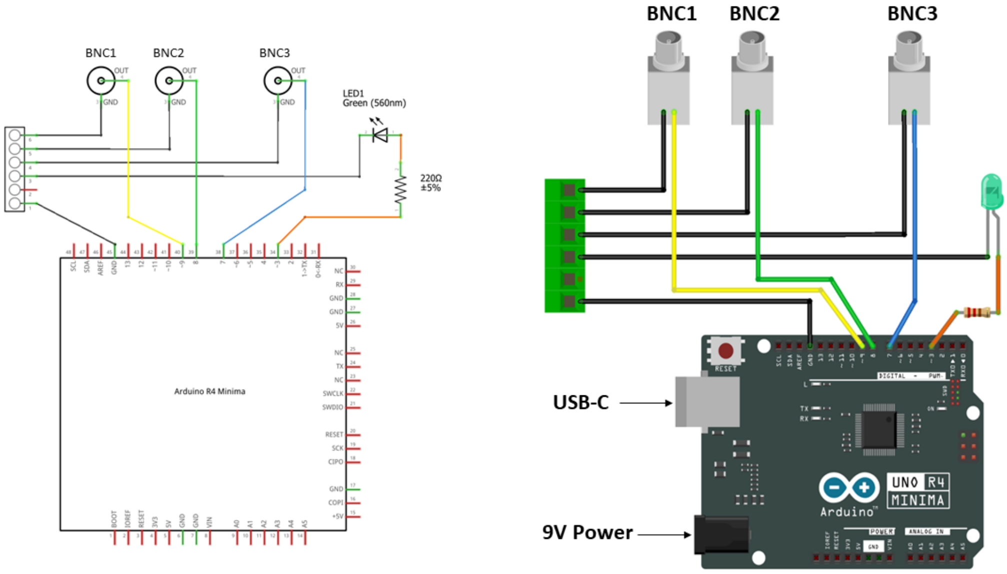

Figure 1 – Silicon Spike's hardware in a.) its schematic circuit, and b.) graphical representation. Full details here: https://ippoz.gitbook.io/siliconspiketriggerbox/.

Eventually, you can use a female BNC instead of a male one, according to your circumstances. Also, you can use the LED color you prefer – and, consequently, the proper resistance. Importantly, in order to grant both data transmission and the square digital wave on the recording, you need to plug the motherboard with both USB-C and 9 Volt 2.1mm power jack.

Once soldered and assembled the above components the device will work properly. However, we suggest building a case (*Fig.* 2) for the device, in order to avoid any potential damage which might hamper its functioning.

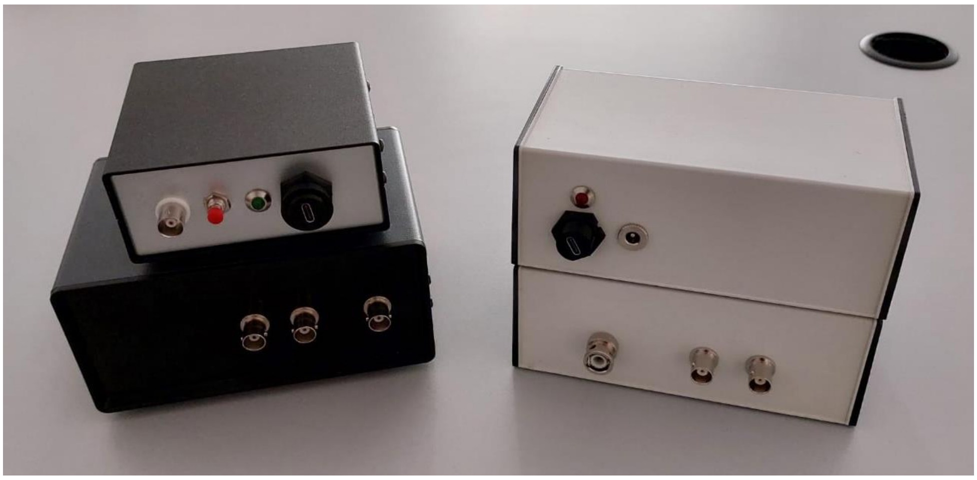

Figure 2 - A few examples of how the circuit can be arranged into a case. Note that their design can be different, since you can flexibly add features according to your needs.

Software

Silicon Spike is composed by two main codes:

- *Communication code* (available here: https://github.com/Ippolz/SiliconSpike/tree/main/Codes/Communication%20Code/MATLAB): is the brief code you use to send information – the stimulation parameters – to the Silicon Spike device. You can copy-paste them, and then just adjust the protocol parameters (e.g., number of TMS pulses; pause between each TMS pulse, etc.) according to your requirements.
- *Main code* (available here: https://github.com/Ippolz/SiliconSpike/tree/main/Codes/Main%20Code/SiliconSpike_MainCode): must be uploaded on the Arduino motherboard, and doesn't need any change. It contains all the instructions to read the stimulation parameters sent through the *communication code*. If the Main code is properly uploaded in the motherboard, the LED will light when the device is powered.

Main code

Once downloaded you just need to open it through the Arduino IDE (see here: https://www.arduino.cc/en/software), and then upload it as it is. This code only needs to be uploaded once.

Communication code

This is the code you will use during each TMS stimulation. You can copy-paste it within your task script to trigger the TMS device without relying on the computer resources. Before running the script, you need to declare the stimulation parameters.

Please note, before starting the code explanation, it is important to find the name of the USB port (“COM”) that you are using with the Silicon Spike device. Here, opening the Device Manager we can see that the Silicon Spike device is connected to port 3, so we declared “COM3”.

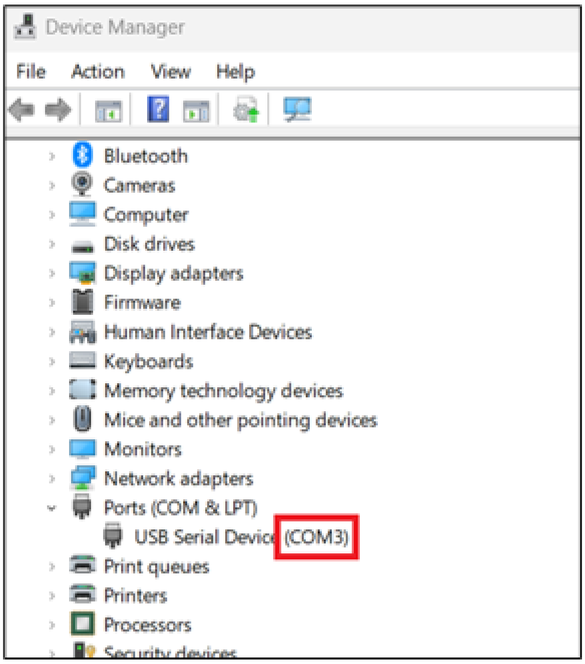

A) *COMMUNICATION CODE*: MATLAB

- **single pulse TMS (spTMS)**

```
% Initialize the serial communication
s = serialport("COM3",115200);
fopen(s);
pause(2);

% Mandatory signature
fwrite(s,"Triggerbox developed by Giuseppe Ippolito:
https://doi.org/10.3758/s13428-025-02653-y");
pause(0.01);

% Declaring marker duration
fwrite(s,"SET,MRK1,3");
pause(0.01);
```

```
13. fwrite(s,"SET,MRK2,5");
14. pause(0.01);
15.
16. % Protocol type (spTMS, dcTMS, rTMS)
17. fwrite(s,"spTMS");
18. pause(0.01);
19.
20. % Commands
21. fwrite(s,"1");
22. fwrite(s,"2");
23.
24. fwrite(s,"A");
25. fwrite(s,"B");
26.
27. % Close the serial communication
28. fwrite(s,"Z");
29. fclose(s);
30. delete(s);
31. clear s;
```

Lines 2-3 initialize the **serial communication**. In older MATLAB versions (prior to 2019b) you can use:

```
% Initialize the serial communication
s = serial ("COM3","BaudRate",115200);
fopen(s);
pause(2);
```

From version 2019b onward is necessary to use:

```
% Initialize the serial communication
s = serialport("COM3",115200);
fopen(s);
pause(2);
```

It is important to change the USB port ("COM") name according to your case as explained above. Also, it is good practice, during the setting phase, to put a *pause* command between each serial string. A 10 ms duration is sufficient in any case, except for the first command, in which the communication between devices is established. In this case, the optimal duration may vary depending on your computer performance. A 2 sec interval should be enough for older devices, too. This pause is not necessary after the setting phase, when delivering TMS pulses.

Then, copy-paste the **mandatory signature**. Without this the following lines won't work.

```
fwrite(s,"Triggerbox developed by Giuseppe Ippolito:
https://doi.org/10.3758/s13428-025-02653-y");
```

After these mandatory steps, you can declare optional stimulation parameters (e.g., IPI, number of pulses, markers) in any order. In this case, since we're going to use a spTMS protocol, the

only parameter we may be interested in is the **voluntary marker** duration. This option allows you to place digital markers of a chosen duration (max 9 different lengths) at any moment. This may be useful in case you need to discriminate between different stimuli's onset (e.g., neural, joy, or fearful faces). Remember that voluntary markers always get delivered through BNC3. Its syntax is:

```
fwrite(s,"SET,MRKN,X");
```

In this case *N* is the preset number (between 1 and 9), and *X* is the marker length. Recalling the two stimuli example, we may want two voluntary markers of different duration (e.g., 3 and 5 ms) to ease our analysis, later. We will write then:

```
fwrite(s,"SET,MRK1,3"); % Preset 1: 3ms marker
fwrite(s,"SET,MRK2,5"); % Preset 2: 5ms marker
```

Our last step before calling the pulse command is declaring which TMS protocol we are going to use. We can choose between spTMS, dcTMS, or rTMS. Since we're using the single pulse one, we will write:

```
fwrite(s,"spTMS");
```

Now that we have declared all of the necessary stimulation parameters, we can finally deliver the TMS pulse. You can independently trigger the BNC1 ("1") or BNC2 ("2"). These lines must be copy-pasted within your code according to your stimulation and task requirements (es. prior to a certain stimulus onset).

```
fwrite(s,"1"); % Trigger BNC1
fwrite(s,"2"); % Trigger BNC2
```

We can also individually call for voluntary markers at any time using letters. Preset 1 (MRK1) is paired with letter "A" – Consequently, preset 2 with "B", 3 with "C", 4 with "D", 5 with "E", 6 with "F", 7 with "G", 8 with "H", and 9 with "I". Since we declared two different markers ("MRK1,3", "MRK2,5") we can trigger them using the letters "A" and "B".

```
fwrite(s,"A"); % Deliver a voluntary marker from preset 1 (3 ms)
fwrite(s,"B"); % Deliver a voluntary marker from preset 2 (5 ms)
```

The command code also includes a line to run in case you may reset any setting and return to the mandatory signature. You can then declare once again the parameter settings. The command is:

```
fwrite(s,"Z"); % Return to the beginning of the setting phase
```

Once the task is completed you can definitely close the serial communication between the Silicon Spike device and the experimental computer, thus avoiding any potential bug.

```
fclose(s);
delete(s);
clear s;
```

- **Dual coil TMS (dcTMS)**

This protocol allows you to trigger pairs of TMS pulses, adding the option to choose the distance in ms between the two pulses. If you've read the previous section (spTMS) most of this information will be redundant, aside from the "SET,IPI" parameter.

```
% Initialize the serial communication
s = serialport("COM3",115200);
fopen(s);
pause(2);

% Mandatory signature
fwrite(s,"Triggerbox developed by Giuseppe Ippolito: https://doi.org/10.3758/s13428-025-02653-y");
pause(0.01);

% Declaring the distance between the two TMS pulses
fwrite(s,"SET,IPI1,30");
pause(0.01);
fwrite(s,"SET,IPI2,50");
pause(0.01);
fwrite(s,"SET,IPI3,70");
pause(0.01);

% Declaring marker duration
fwrite(s,"SET,MRK1,3");
pause(0.01);
fwrite(s,"SET,MRK2,5");
pause(0.01);
fwrite(s,"SET,MRK3,7");
pause(0.01);

% Protocol type (spTMS, dcTMS, rTMS)
fwrite(s,"dcTMS");
pause(0.01);

% Commands
fwrite(s,"1");
fwrite(s,"2");
fwrite(s,"3");

fwrite(s,"A");
fwrite(s,"B");
fwrite(s,"C");

% Close the serial communication
fwrite(s,"Z");
fclose(s);
delete(s);
```

```
43. clear s;
```

Lines 2-3 initialize the **serial communication**. In older MATLAB versions (prior to 2019b) you can use:

```
% Initialize the serial communication
s = serial ("COM3","BaudRate",115200);
fopen(s);
pause(2);
```

From version 2019b onward is necessary to use:

```
% Initialize the serial communication
s = serialport("COM3",115200);
fopen(s);
pause(2);
```

It is important to change the USB port (“COM”) name according to your case as explained above. Also, it is good practice, during the setting phase, to put a *pause* command between each serial string. A 10 ms duration is sufficient in any case, except for the first command, in which the communication between devices is established. In this case, the optimal duration may vary depending on your computer performance. A 2 sec interval should be enough for older devices, too. This pause is not necessary after the setting phase, when delivering TMS pulses.

Then, copy-paste the **mandatory signature**. Without this the following lines won’t work.

```
fwrite(s,"Triggerbox developed by Giuseppe Ippolito:
https://doi.org/10.3758/s13428-025-02653-y");
```

After these mandatory steps, you can declare optional stimulation parameters (e.g., IPI, number of pulses, markers) in any order. In this case, since we’re going to use a spTMS protocol, the only parameter we may be interested in is the **voluntary marker** duration. This option allows you to place digital markers of a chosen duration (max 9 different lengths) at any moment. This may be useful in case you need to discriminate between different stimuli’s onset (e.g., neural, joy, or fearful faces). Remember that voluntary markers always get delivered through BNC3. Its syntax is:

```
fwrite(s,"SET,MRKN,X");
```

In this case *N* is the preset number (between 1 and 9), and *X* is the marker length. Recalling the three stimuli example, we may want three voluntary markers of different duration (e.g., 3, 5, and 7 ms) ease our analysis, later. We will write then:

```
fwrite(s,"SET,MRK1,3"); % Preset 1: 3ms marker
fwrite(s,"SET,MRK2,5"); % Preset 2: 5ms marker
fwrite(s,"SET,MRK3,7"); % Preset 3: 7ms marker
```

For dcTMS paradigms it is also important to declare the distance in ms between the two pulses (IPI), delivered from BNC1 and then BNC2. Its syntax is:

```
fwrite(s,"SET,IPIN,X");
```

In this case N is the preset number (between 1 and 9), and X is the distance between pulses in ms. For example, we may want to test the difference in delivering pulse pairs with different latencies when the experimental stimulus occurs. We will write then:

```
fwrite(s,"SET,IPI1,30"); % Preset 1: 30ms distance
fwrite(s,"SET,IPI2,50"); % Preset 2: 50ms distance
fwrite(s,"SET,IPI3,70"); % Preset 3: 70ms distance
```

Our last step before calling the pulse command is declaring which TMS protocol we are going to use. We can choose between spTMS, dcTMS, or rTMS. Since we're using the dual coil one, we will write:

```
fwrite(s,"dcTMS");
```

Now that we have declared all of the necessary stimulation parameters, we can finally trigger the TMS device. You can independently trigger each of the declared protocols. These lines must be copy-pasted within your code according to your stimulation and task requirements (es. prior to a certain stimulus onset).

```
fwrite(s,"1"); % Trigger protocol 1 (two pulses with a 30 ms distance)
fwrite(s,"2"); % Trigger protocol 2 (two pulses with a 50 ms distance)
fwrite(s,"3"); % Trigger protocol 3 (two pulses with a 70 ms distance)
```

We can also individually call for voluntary markers at any time using letters. Preset 1 (MRK1) is paired with letter "A" – Consequently, preset 2 with "B", 3 with "C", 4 with "D", 5 with "E", 6 with "F", 7 with "G", 8 with "H", and 9 with "I". Since we declared three different markers ("MRK1,3", "MRK2,5", "MRK3,7") we can trigger them using the letters "A", "B", and "C".

```
fwrite(s,"A"); % Deliver a voluntary marker from preset 1 (3 ms)
fwrite(s,"B"); % Deliver a voluntary marker from preset 2 (5 ms)
fwrite(s,"C"); % Deliver a voluntary marker from preset 3 (7 ms)
```

The command code also includes a line to run in case you may reset any setting and return to the mandatory signature. You can then declare once again the parameter settings. The command is:

```
fwrite(s,"Z"); % Return to the beginning of the setting phase
```

Once the task is completed you can definitely close the serial communication between the Silicon Spike device and the experimental computer, thus avoiding any potential bug.

```
fclose(s);
delete(s);
```

```
clear s;
```

- **repetitive TMS (rTMS)**

This protocol allows you to trigger trains of TMS pulses, adding the option to choose the distance in ms between the two pulses and the number of pulses within each train. If you've read the previous section (dcTMS) most of this information will be redundant, aside from the "SET,nPULS" parameter.

```
% Initialize the serial communication
s = serialport("COM3",115200);
fopen(s);
pause(2);

% Mandatory signature
fwrite(s,"Triggerbox developed by Giuseppe Ippolito: https://doi.org/10.3758/s13428-025-02653-y");
pause(0.01);

% Declaring the distance between each TMS pulse
fwrite(s,"SET,IPI1,80");
pause(0.01);
fwrite(s,"SET,IPI2,100");
pause(0.01);
fwrite(s,"SET,IPI3,120");
pause(0.01);

% Declaring the number of pulses within each train
fwrite(s,"SET,nPULS1,4");
pause(0.01);
fwrite(s,"SET,nPULS2,5");
pause(0.01);
fwrite(s,"SET,nPULS3,6");
pause(0.01);

% Declaring marker duration
fwrite(s,"SET,MRK1,3");
pause(0.01);
fwrite(s,"SET,MRK2,5");
pause(0.01);
fwrite(s,"SET,MRK3,7");
pause(0.01);

% Protocol type (spTMS, dcTMS, rTMS)
fwrite(s,"rTMS");
pause(0.01);

```

```
38. % Commands
39. fwrite(s,"1");
40. fwrite(s,"2");
41. fwrite(s,"3");
42.
43. fwrite(s,"A");
44. fwrite(s,"B");
45. fwrite(s,"C");
46.
47. % Close the serial communication
48. fwrite(s,"Z");
49. fclose(s);
50. delete(s);
51. clear s;
```

Lines 2-3 initialize the **serial communication**. In older MATLAB versions (prior to 2019b) you can use:

```
% Initialize the serial communication
s = serial ("COM3","BaudRate",115200);
fopen(s);
pause(2);
```

From version 2019b onward is necessary to use:

```
% Initialize the serial communication
s = serialport("COM3",115200);
fopen(s);
pause(2);
```

It is important to change the USB port ("COM") name according to your case as explained above. Also, it is good practice, during the setting phase, to put a *pause* command between each serial string. A 10 ms duration is sufficient in any case, except for the first command, in which the communication between devices is established. In this case, the optimal duration may vary depending on your computer performance. A 2 sec interval should be enough for older devices, too. This pause is not necessary after the setting phase, when delivering TMS pulses.

Then, copy-paste the **mandatory signature**. Without this the following lines won't work.

```
fwrite(s,"Triggerbox developed by Giuseppe Ippolito:
https://doi.org/10.3758/s13428-025-02653-y");
```

After these mandatory steps, you can declare optional stimulation parameters (e.g., IPI, number of pulses, markers) in any order. In this case, since we're going to use a spTMS protocol, the only parameter we may be interested in is the **voluntary marker** duration. This option allows you to place digital markers of a chosen duration (max 9 different lengths) at any moment. This may be useful in case you need to discriminate between different stimuli's onset (e.g., neural,

joy, or fearful faces). Remember that voluntary markers always get delivered through BNC3. Its syntax is:

```
fwrite(s,"SET,MRKN,X");
```

In this case *N* is the preset number (between 1 and 9), and *X* is the marker length. Recalling the three stimuli example, we may want three voluntary markers of different duration (e.g., 3, 5, and 7 ms) ease our analysis, later. We will write then:

```
fwrite(s,"SET,MRK1,3"); % Preset 1: 3ms marker
fwrite(s,"SET,MRK2,5"); % Preset 2: 5ms marker
fwrite(s,"SET,MRK3,7"); % Preset 3: 7ms marker
```

For rTMS paradigms it is important to declare the number of pulses constituting each train, triggered simultaneously from BNC1 and BNC2. Its syntax is:

```
fwrite(s,"SET,nPULSN,X");
```

In this case N is the preset number (between 1 and 9), and X is the number of pulses within the train. For example, we may want to test the difference in delivering trains of 4, 5, or 6 pulses prior to the stimulus occurrence. We will write then:

```
fwrite(s,"SET,nPULS1,4"); % Preset 1: 4 pulses in each train
fwrite(s,"SET,nPULS2,5"); % Preset 2: 5 pulses in each train
fwrite(s,"SET,nPULS3,6"); % Preset 3: 6 pulses in each train
```

Additionally, in rTMS paradigms it is also important to declare the distance in ms between the two pulses (IPI), delivered from BNC1 and then BNC2. Its syntax is:

```
fwrite(s,"SET,IPIN,X");
```

In this case N is the preset number (between 1 and 9), and X is the distance between pulses in ms. For example, we may want an 80 ms interval in the first preset (4 pulses), a 100 ms interval for the second one (5 pulses), and a 120 ms interval for the third one (6 pulses). We will write then:

```
fwrite(s,"SET,IPI1,80");  % Preset 1: 80ms distance
fwrite(s,"SET,IPI2,100"); % Preset 2: 100ms distance
fwrite(s,"SET,IPI3,120"); % Preset 3: 120ms distance
```

Our last step before calling the pulse command is declaring which TMS protocol we are going to use. We can choose between spTMS, dcTMS, or rTMS. Since we're using the double pulse one, we will write:

```
fwrite(s,"rTMS");
```

Now that we have declared all of the necessary stimulation parameters, we can finally trigger the TMS device. You can independently trigger each of the declared protocols. These lines

must be copy-pasted within your code according to your stimulation and task requirements (es. prior to a certain stimulus onset).

```
fwrite(s,"1"); % Protocol 1 (one train of 4 pulses with a 80ms distance)
fwrite(s,"2"); % Protocol 2 (one train of 5 pulses with a 100ms distance)
fwrite(s,"3"); % Protocol 3 (one train of 6 pulses with a 120ms distance)
```

We can also individually call for voluntary markers at any time using letters. Preset 1 (MRK1) is paired with letter "A" – Consequently, preset 2 with "B", 3 with "C", 4 with "D", 5 with "E", 6 with "F", 7 with "G", 8 with "H", and 9 with "I". Since we declared three different markers ("MRK1,3", "MRK2,5", "MRK3,7") we can trigger them using the letters "A", "B", and "C".

```
fwrite(s,"A"); % Deliver a voluntary marker from preset 1 (3 ms)
fwrite(s,"B"); % Deliver a voluntary marker from preset 2 (5 ms)
fwrite(s,"C"); % Deliver a voluntary marker from preset 3 (7 ms)
```

The command code also includes a line to run in case you may reset any setting and return to the mandatory signature. You can then declare once again the parameter settings. The command is:

```
fwrite(s,"Z"); % Return to the beginning of the setting phase
```

Once the task is completed you can definitely close the serial communication between the Silicon Spike device and the experimental computer, thus avoiding any potential bug.

```
fclose(s);
delete(s);
clear s;
```

B) *COMMUNICATION CODE*: PYTHON

- **single pulse TMS (spTMS)**

```
# Import the required packages
from serial import Serial, SerialException

# Initialize the serial communication
s = Serial()
s.port = "COM3"
s.baudrate = 115200
s.open()

# Mandatory signature
s.write(b"Triggerbox developed by Giuseppe Ippolito: https://doi.org/10.3758/s13428-025-02653-y\n")

# For declaring marker duration
s.write(b"SET,MRK1,3\n")
```

```
13.    s.write(b"SET,MRK2,5\n")
14.    s.write(b"SET,MRK3,7\n")

15.    # Protocol type (rTMS, dcTMS, spTMS)
16.    s.write(b"spTMS\n")

17.    # Commands
18.    s.write(b"1\n")
19.    s.write(b"2\n")
20.    s.write(b"3\n")

21.    s.write(b"A\n")
22.    s.write(b"B\n")
23.    s.write(b"C\n")

24.    # Close the serial communication
25.    s.write(b"Z\n")
26.    s.close()
27.    del s
```

Lines 4-7 initialize the **serial communication**. Python can use:

```
s = Serial()
s.port = "COM3"
s.baudrate = 115200
s.open()
```

as the same as:

```
s = serial.Serial(port = "COM3", baudrate = 115200)
s.open()
```

It is important to change the USB port ("COM") name according to your case as explained above.

Then, copy-paste the **mandatory signature**. Without this the following lines won't work.

```
s.write(b"Triggerbox developed by Giuseppe Ippolito:
https://doi.org/10.3758/s13428-025-02653-y\n")
```

After these mandatory steps, you can declare optional stimulation parameters (e.g., IPI, number of pulses, markers) in any order. In this case, since we're going to use a spTMS protocol, the only parameter we may be interested in is the **voluntary marker** duration. This option allows you to place digital markers of a chosen duration (max 9 different lengths) at any moment. This may be useful in case you need to discriminate between different stimuli's onset (e.g., neural, joy, or fearful faces). Remember that voluntary markers always get delivered through BNC3. Its syntax is:

```
s.write(b"SET,MRK\n")
```

In this case *N* is the preset number (between 1 and 9), and *X* is the marker length. Recalling the two stimuli example, we may want three voluntary markers of different duration (e.g., 3 and 5 ms) to ease our analysis, later. We will write then:

```
s.write(b"SET,MRK1,3\n") # Preset 1: 3ms marker
s.write(b"SET,MRK2,5\n") # Preset 2: 5ms marker
```

Our last step before calling the pulse command is declaring which TMS protocol we are going to use. We can choose between spTMS, dcTMS, or rTMS. Since we're using the single pulse one, we will write:

```
s.write(b"spTMS\n")
```

Now that we have declared all of the necessary stimulation parameters, we can finally deliver the TMS pulse. You can independently trigger the BNC1 ("1") or BNC2 ("2"). These lines must be copy-pasted within your code according to your stimulation and task requirements (es. prior to a certain stimulus onset).

```
s.write(b"1\n") # Trigger BNC1
s.write(b"2\n") # Trigger BNC2
```

We can also individually call for voluntary markers at any time using letters. Preset 1 (MRK1) is paired with letter "A" – Consequently, preset 2 with "B", 3 with "C", 4 with "D", 5 with "E", 6 with "F", 7 with "G", 8 with "H", and 9 with "I". Since we declared two different markers ("MRK1,3", "MRK2,5") we can trigger them using the letters "A" and "B".

```
s.write(b"A\n") # Deliver a voluntary marker from preset 1 (3 ms)
s.write(b"B\n") # Deliver a voluntary marker from preset 2 (5 ms)
```

The command code also includes a line to run in case you may reset any setting and return to the mandatory signature. You can then declare once again the parameter settings. The command is:

```
s.write(b"Z\n") # Return to the beginning of the setting phase
```

Once the task is completed you can definitely close the serial communication between the Silicon Spike device and the experimental computer, thus avoiding any potential bug.

```
s.close()
del s
```

- **Dual coil TMS (dcTMS)**

This protocol allows you to trigger pairs of TMS pulses, adding the option to choose the distance in ms between the two pulses. If you've read the previous section (spTMS) most of this information will be redundant, aside from the "SET,IPI" parameter.

```
# Import the required packages
```

```
2. from serial import Serial, SerialException

3. # Initialize the serial communication
4. s = Serial()
5. s.port = "COM3"
6. s.baudrate = 115200
7. s.open()

8. # Mandatory signature
9. s.write(b"Triggerbox developed by Giuseppe Ippolito:
   https://doi.org/10.3758/s13428-025-02653-y\n")

10.   #  For placing markers
11.   s.write(b"SET,MRKN\n")

12.   # For declaring marker duration
13.   s.write(b"SET,IPI1,30\n")
14.   s.write(b"SET,IPI2,50\n")
15.   s.write(b"SET,IPI3,70\n")

16.   # For declaring marker duration
17.   s.write(b"SET,MRK1,3\n")
18.   s.write(b"SET,MRK2,5\n")
19.   s.write(b"SET,MRK3,7\n")

20.   # Protocol type (rTMS, dcTMS, spTMS)
21.   s.write(b"dcTMS\n")

22.   # Commands
23.   s.write(b"1\n")
24.   s.write(b"2\n")
25.   s.write(b"3\n")

26.   s.write(b"A\n")
27.   s.write(b"B\n")
28.   s.write(b"C\n")

29.   # Close the serial communication
30.   s.write(b"Z\n")
31.   s.close()
32.   del s
```

Lines 4-7 initialize the **serial communication**. Python can use:

```
s = Serial()
s.port = "COM3"
s.baudrate = 115200
s.open()
```

as the same as:

```
s = serial.Serial(port = "COM3", baudrate = 115200)
s.open()
```

It is important to change the USB port ("COM") name according to your case as explained above.

Then, copy-paste the **mandatory signature**. Without this the following lines won't work.

```
s.write(b"Triggerbox developed by Giuseppe Ippolito:
https://doi.org/10.3758/s13428-025-02653-y\n")
```

After these mandatory steps, you can declare optional stimulation parameters (e.g., IPI, number of pulses, markers) in any order. In this case, since we're going to use a spTMS protocol, the only parameter we may be interested in is the **voluntary marker** duration. This option allows you to place digital markers of a chosen duration (max 9 different lengths) at any moment. This may be useful in case you need to discriminate between different stimuli's onset (e.g., neural, joy, or fearful faces). Remember that voluntary markers always get delivered through BNC3. Its syntax is:

```
s.write(b"SET,MRKN,X\n")
```

In this case *N* is the preset number (between 1 and 9), and *X* is the marker length. Recalling the three stimuli example, we may want three voluntary markers of different duration (e.g., 3, 5, and 7 ms) ease our analysis, later. We will write then:

```
s.write(b"SET,MRK1,3\n") # Preset 1: 3ms marker
s.write(b"SET,MRK2,5\n") # Preset 2: 5ms marker
s.write(b"SET,MRK3,7\n") # Preset 3: 7ms marker
```

For dcTMS paradigms it is also important to declare the distance in ms between the two pulses (IPI), delivered from BNC1 and then BNC2. Its syntax is:

```
s.write(b"SET,IPIN,X\n")
```

In this case N is the preset number (between 1 and 9), and X is the distance between pulses in ms. For example, we may want to test the difference in delivering pulse pairs with different latencies when the experimental stimulus occurs. We will write then:

```
s.write(b"SET,IPI1,30\n") # Preset 1: 30ms distance
s.write(b"SET,IPI2,50\n") # Preset 2: 50ms distance
s.write(b"SET,IPI3,70\n") # Preset 3: 70ms distance
```

Our last step before calling the pulse command is declaring which TMS protocol we are going to use. We can choose between spTMS, dcTMS, or rTMS. Since we're using the double pulse one, we will write:

```
s.write(b"dcTMS\n")
```

Now that we have declared all of the necessary stimulation parameters, we can finally trigger the TMS device. You can independently trigger each of the declared protocols. These lines must be copy-pasted within your code according to your stimulation and task requirements (es. prior to a certain stimulus onset).

```
s.write(b"1\n")  # Trigger protocol 1 (two pulses with a 30 ms distance)
s.write(b"2\n")  # Trigger protocol 2 (two pulses with a 50 ms distance)
s.write(b"3\n")  # Trigger protocol 3 (two pulses with a 70 ms distance)
```

We can also individually call for voluntary markers at any time using letters. Preset 1 (MRK1) is paired with letter "A" – Consequently, preset 2 with "B", 3 with "C", 4 with "D", 5 with "E", 6 with "F", 7 with "G", 8 with "H", and 9 with "I". Since we declared three different markers ("MRK1,3", "MRK2,5", "MRK3,7") we can trigger them using the letters "A", "B", and "C".

```
s.write(b"A\n") # Deliver a voluntary marker from preset 1 (3 ms)
s.write(b"B\n") # Deliver a voluntary marker from preset 2 (5 ms)
s.write(b"C\n") # Deliver a voluntary marker from preset 3 (7 ms)
```

The command code also includes a line to run in case you may reset any setting and return to the mandatory signature. You can then declare once again the parameter settings. The command is:

```
s.write(s,"Z\n") # Return to the beginning of the setting phase
```

Once the task is completed you can definitely close the serial communication between the Silicon Spike device and the experimental computer, thus avoiding any potential bug.

```
s.close()
del s
```

- **repetitive TMS (rTMS)**

This protocol allows you to trigger trains of TMS pulses, adding the option to choose the distance in ms between the two pulses and the number of pulses within each train. If you've read the previous section (dcTMS) most of this information will be redundant, aside from the "SET,nPULS" parameter.

```
# Import the required packages
from serial import Serial, SerialException

# Initialize the serial communication
s = Serial()
s.port = "COM3"
s.baudrate = 115200
s.open()

# Mandatory signature
s.write(b"Triggerbox developed by Giuseppe Ippolito: https://doi.org/10.3758/s13428-025-02653-y\n")

# For placing markers
s.write(b"SET,MRK\n")
```

```
12.    # For declaring the distance between the two TMS pulses
13.    s.write(b"SET,IPI1,80\n")
14.    s.write(b"SET,IPI2,100\n")
15.    s.write(b"SET,IPI3,120\n")

16.    # For declaring the number of pulses within each train
17.    s.write(b"SET,nPULS1,5\n")
18.    s.write(b"SET,nPULS2,5\n")
19.    s.write(b"SET,nPULS3,5\n")

20.    # For declaring marker duration
21.    s.write(b"SET,MRK1,3\n")
22.    s.write(b"SET,MRK2,5\n")
23.    s.write(b"SET,MRK3,7\n")

24.    # Protocol type (rTMS, dcTMS, spTMS)
25.    s.write(b"rTMS\n")

26.    # Commands
27.    s.write(b"1\n")
28.    s.write(b"2\n")
29.    s.write(b"3\n")

30.    s.write(b"A\n")
31.    s.write(b"B\n")
32.    s.write(b"C\n")

33.    # Close the serial communication
34.    s.write(b"Z\n")
35.    s.close()
36.    del s
```

Lines 4-7 initialize the **serial communication**. Python can use:

```
s = Serial()
s.port = "COM3"
s.baudrate = 115200
s.open()
```

as the same as:

```
s = serial.Serial(port = "COM3", baudrate = 115200)
s.open()
```

It is important to change the USB port ("COM") name according to your case as explained above.

Then, copy-paste the **mandatory signature**. Without this the following lines won't work.

```
s.write(b"Triggerbox developed by Giuseppe Ippolito:
https://doi.org/10.3758/s13428-025-02653-y\n")
```

After these mandatory steps, you can declare optional stimulation parameters (e.g., IPI, number of pulses, markers) in any order. In this case, since we're going to use a spTMS protocol, the only parameter we may be interested in is the **voluntary marker** duration. This option allows you to place digital markers of a chosen duration (max 9 different lengths) at any moment. This may be useful in case you need to discriminate between different stimuli's onset (e.g., neural, joy, or fearful faces). Remember that voluntary markers always get delivered through BNC3. Its syntax is:

```
s.write(b"SET,MRKN,X\n")
```

In this case *N* is the preset number (between 1 and 9), and *X* is the marker length. Recalling the three stimuli example, we may want three voluntary markers of different duration (e.g., 3, 5, and 7 ms) ease our analysis, later. We will write then:

```
s.write(b"SET,MRK1,3\n") # Preset 1: 3ms marker
s.write(b"SET,MRK2,5\n") # Preset 2: 5ms marker
s.write(b"SET,MRK3,7\n") # Preset 3: 7ms marker
```

For rTMS paradigms it is important to declare the number of pulses constituting each train, triggered simultaneously from BNC1 and BNC2. Its syntax is:

```
s.write(b"SET,nPULSN,X\n")
```

In this case N is the preset number (between 1 and 9), and X is the number of pulses within the train. For example, we may want to test the difference in delivering trains of 4, 5, or 6 pulses prior to the stimulus occurrence. We will write then:

```
s.write(b"SET,nPULS1,4\n") # Preset 1: 4 pulses in each train
s.write(b"SET,nPULS2,5\n") # Preset 2: 5 pulses in each train
s.write(b"SET,nPULS3,6\n") # Preset 3: 6 pulses in each train
```

Additionally, in rTMS paradigms it is also important to declare the distance in ms between the two pulses (IPI), delivered from BNC1 and then BNC2. Its syntax is:

```
s.write(b"SET,IPIN,X\n")
```

In this case N is the preset number (between 1 and 9), and X is the distance between pulses in ms. For example, we may want an 80 ms interval in the first preset (4 pulses), a 100 ms interval for the second one (5 pulses), and a 120 ms interval for the third one (6 pulses). We will write then:

```
s.write(b"SET,IPI1,80\n")  # Preset 1: 80ms distance
s.write(b"SET,IPI2,100\n") # Preset 2: 100ms distance
s.write(b"SET,IPI3,120\n") # Preset 3: 120ms distance
```

Our last step before calling the pulse command is declaring which TMS protocol we are going to use. We can choose between spTMS, dcTMS, or rTMS. Since we're using the double pulse one, we will write:

```
s.write(b"rTMS\n")
```

Now that we have declared all of the necessary stimulation parameters, we can finally trigger the TMS device. You can independently trigger each of the declared protocols. These lines must be copy-pasted within your code according to your stimulation and task requirements (es. prior to a certain stimulus onset).

```
s.write(b"1\n") # Protocol 1 (one train of 4 pulses with a 80ms distance)
s.write(b"2\n") # Protocol 2 (one train of 5 pulses with a 100ms distance)
s.write(b"3\n") # Protocol 3 (one train of 6 pulses with a 120ms distance)
```

We can also individually call for voluntary markers at any time using letters. Preset 1 (MRK1) is paired with letter "A" – Consequently, preset 2 with "B", 3 with "C", 4 with "D", 5 with "E", 6 with "F", 7 with "G", 8 with "H", and 9 with "I". Since we declared three different markers ("MRK1,3", "MRK2,5", "MRK3,7") we can trigger them using the letters "A", "B", and "C".

```
s.write(b"A\n") # Deliver a voluntary marker from preset 1 (3 ms)
s.write(b"B\n") # Deliver a voluntary marker from preset 2 (5 ms)
s.write(b"C\n") # Deliver a voluntary marker from preset 3 (7 ms)
```

The command code also includes a line to run in case you may reset any setting and return to the mandatory signature. You can then declare once again the parameter settings. The command is:

```
s.write(b"Z\n") # Return to the beginning of the setting phase
```

Once the task is completed you can definitely close the serial communication between the Silicon Spike device and the experimental computer, thus avoiding any potential bug.

```
s.close()
del s
```
